# Moderate (20%) fructose‐enriched diet stimulates salt‐sensitive hypertension with increased salt retention and decreased renal nitric oxide

**DOI:** 10.14814/phy2.13162

**Published:** 2017-04-13

**Authors:** Kevin L. Gordish, Kamal M. Kassem, Pablo A. Ortiz, William H. Beierwaltes

**Affiliations:** ^1^ Department of PhysiologyWayne State School of MedicineDetroitMichigan; ^2^ Department of Internal MedicineHypertension and Vascular Research DivisionHenry Ford HospitalDetroitMichigan

**Keywords:** Fructose, glucose, nitric oxide, sodium balance, sodium excretion

## Abstract

Previously, we reported that 20% fructose diet causes salt‐sensitive hypertension. In this study, we hypothesized that a high salt diet supplemented with 20% fructose (in drinking water) stimulates salt‐sensitive hypertension by increasing salt retention through decreasing renal nitric oxide. Rats in metabolic cages consumed normal rat chow for 5 days (baseline), then either: (1) normal salt for 2 weeks, (2) 20% fructose in drinking water for 2 weeks, (3) 20% fructose for 1 week, then fructose + high salt (4% NaCl) for 1 week, (4) normal chow for 1 week, then high salt for 1 week, (5) 20% glucose for 1 week, then glucose + high salt for 1 week. Blood pressure, sodium excretion, and cumulative sodium balance were measured. Systolic blood pressure was unchanged by 20% fructose or high salt diet. 20% fructose + high salt increased systolic blood pressure from 125 ± 1 to 140 ± 2 mmHg (*P *< 0.001). Cumulative sodium balance was greater in rats consuming fructose + high salt than either high salt, or glucose + high salt (114.2 ± 4.4 vs. 103.6 ± 2.2 and 98.6 ± 5.6 mEq/Day19; *P *< 0.05). Sodium excretion was lower in fructose + high salt group compared to high salt only: 5.33 ± 0.21 versus 7.67 ± 0.31 mmol/24 h; *P *< 0.001). Nitric oxide excretion was 2935 ± 256 *μ*mol/24 h in high salt‐fed rats, but reduced by 40% in the 20% fructose + high salt group (2139 ± 178 *μ*mol /24 hrs *P *< 0.01). Our results suggest that fructose predisposes rats to salt‐sensitivity and, combined with a high salt diet, leads to sodium retention, increased blood pressure, and impaired renal nitric oxide availability.

## Introduction

Consumption of high‐fructose corn syrup as a food sweetener has increased dramatically in the American diet since its introduction in the early 1970s due to its low cost. Consuming large quantities of fructose has been implicated in the American epidemic of diabetes (Johnson et al. 2007; Nakagawa et al. 2006; Schulze et al. 2004) metabolic syndrome (Dhingra et al. 2007), obesity (Angelopoulos et al. 2009; Ludwig et al. 2001; Teff et al. 2004), renal failure (Gersch et al. 2007; Sánchez‐Lozada et al. 2007), and hypertension (Chou et al. 2011; Hwang et al. 1987; Nakayama et al. 2010) including salt‐sensitive hypertension (Nishimoto et al. 2002; Sechi 1999). While it is clear that the consumption of very high levels of fructose is detrimental to health, it is less clear whether consumption of fructose more relevant to human consumption (10–20% of caloric intake) is harmful to health. It is also unclear how the renal actions of fructose differ from those of glucose. Is it just a function of “added sugar” in the diet (Johnson et al. 2009; Yang et al. 2014), or does fructose have unique characteristics associated with these pathologies?

Persistently elevated high fructose, but not glucose intake, has been associated with development of hypertension, followed by type‐2 diabetes and metabolic syndrome (Tran et al. 2009). A rat model of fructose‐induced hypertension and diabetes was first reported by Hwang et al. (1987)). However, the role of the kidney in the development of fructose‐induced hypertension is not well understood. In this model, several metabolic abnormalities develop after 2 weeks of very high fructose intake (40–65%), including hypertension. However, when lower levels of fructose (20% of caloric intake) are used that more closely reflect the upper levels of the human diet (Mamikutty et al. 2014), the increase in blood pressure takes longer to develop (around 8 weeks), and this is after leptin or insulin‐resistance develop. Increased dietary fructose intake has also been reported to stimulate both salt and fructose absorption in the small intestine and in the renal proximal tubule (Soleimani 2011) through coordinated activation of sugar transporters and the sodium hydrogen exchanger, NHE3. A high fructose diet results in decreased salt excretion (Singh et al. 2008) but whether this is the result of sodium retention is not clear. In contrast, a low salt diet has been reported to blunt fructose‐induced hypertension (Catena et al. 2003).

How elevated fructose intake is linked to increased blood pressure remains an important question. However, the role of the renin‐angiotensin system, which is a key regulator of blood pressure and Na excretion (Castrop et al. 2010), cannot be discounted. High circulating angiotensin II has been shown to cause both salt‐independent and dependent forms of hypertension. Angiotensin receptor antagonists and renin inhibitors reduce fructose‐induced hypertension (Chou et al. 2011, 2013; Farah et al. 2007). However, a high salt diet should suppress plasma renin activity (PRA) thereby diminishing the influence of Ang II.

Renal perfusion is, in part controlled by a balance of endothelial nitric oxide (NO) synthesis and endogenous reactive oxygen species production (Gordish and Beierwaltes 2014). There is substantial literature regarding nitric oxide deficiency and/or oxidative stress that may contribute to hypertension (Wilcox 2005). Ingestion of 60% fructose for over 8 weeks (Behr‐Roussel et al. 2008), or 10% over 12 weeks (El‐Bassossy and Watson 2015), has been reported to increase 8‐isoprostane excretion, a marker for renal reactive oxygen species production. High salt diets have been shown to increase urinary nitric oxide excretion (Shultz and Tolins 1993). This might reflect a compensatory response to account for increased sodium excretion to reduce the salt load and help maintain normal blood pressure. Could fructose undermine this compensatory response?

Historically, sodium balance studies performed on spontaneous hypertensive rats demonstrated the rapid onset of hypertension was linked to increased sodium retention (Beierwaltes et al. 1982). Thus, we proposed similar studies to see if 20% fructose intake would promote salt‐sensitive hypertension. We expected, based on our previous data (Cabral et al. 2014), that 20% fructose ingestion coupled with a 4% high salt diet would induce a salt‐sensitive hypertension earlier than it occurs with 20% fructose alone, and hypothesized this is due to increased sodium retention prior to the development of diabetes or metabolic syndrome. We also proposed that fructose‐linked increases in blood pressure are in part due to impaired nitric oxide synthesis and an altered sensitivity of plasma renin activity (PRA) to high salt ingestion.

## Methods

Male Sprague‐Dawley rats weighing 200–225 g were housed in standard caging. Rats were fed normal rat chow containing 0.4% sodium chloride (Harlan Teklad, Madison, WI) and allowed free access to distilled water. Prior to the beginning of the protocol, rats were pretrained on a non‐invasive tail cuff plethsmography multi‐channel system (Kent Scientific, Torrington, CT) three times a week for 2 weeks at the same time of day to measure systolic pressure. This training assures familiarity of the rats with the system, reduces possible stress and develops consistency in sequential readings. Once trained, the experimental protocols commenced using the exact same methods. Tail cuff methodology in rats only produces a measurement of systolic blood pressure, and does not provide an accurate measurement of mean blood pressure, though in our experience (Beierwaltes et al. 1982; Cabral et al. 2014) changes in systolic reflect changes in mean blood pressure. All procedures were approved by the Henry Ford Health System Institutional Animal Care and Use Committee (IACUC) and adhered to the guiding principles in the care and use of experimental animals in accordance with the National Institute of Health (NIH) guidelines. Henry Ford Hospital operates an AALAC‐certified animal care facility.

### Protocol 1: Blood pressure and cumulative sodium balance in response to fructose and a high salt diet with paired‐feeding to control sodium intake

#### Diet assignment

After the initial training period, rats (weighing in the low 300 g range) were moved to metabolic caging and allowed to acclimate to the new environment for 1 day. In the first protocol (sodium balance; pair feeding), we employed five different dietary groups of rats over 2 weeks of treatment after obtaining baseline values. These included group 1, control (C); given distilled water and fed a normal rat chow (*n *= 9); group 2, given 20% fructose (F) in the drinking water and fed normal rat chow (*n *= 9); group 3, given 20% fructose in the drinking water and fed high salt (F+HS) rat chow (4% NaCl) during the second week (*n *= 18); group 4, given distilled water and fed high salt (HS) rat chow during the second week (*n *= 9), and group 5, given 20% glucose and fed high salt (G+HS) rat chow during the second week (*n *= 8). Both the normal (0.4% NaCl) and high salt (4% NaCl) solid diets were obtained from Harlan Teklad (Madison WI), and otherwise were identical in nutrient and caloric content.

The experimental protocol involved 2 weeks of study. All rats were pair‐fed normal rat chow containing 0.4% NaCl (Harlan Teklad, Madison, WI) and allowed free access to distilled water for 5 days for baseline measurements. We used paired‐feeding of solid rat chow to control and maintain constant nutrient and sodium intake under both normal and high salt protocols. During week 1, the experimental diets were initiated with the addition of drinking water containing either 20% fructose or 20% glucose (Sigma Aldrich, St. Louis) to replace distilled water in the F, F + HS, and G + HS Groups. These solutions were prepared freshly every few days to reduce risk of microbial growth, and solutions were stored in a cold room. During week 2, the F + HS, HS and G+HS groups were switched from normal rat chow to the 4% NaCl diet.

Every effort was taken to ensure food/caloric consumption of rat chow was equal between groups by pair feeding. During the course of the protocol, rat chow was rationed equally to all groups, and increased over time to accommodate growth. Rat chow was weighed daily to the nearest tenth of a gram and directly placed into each cage. Rat chow was restricted across all five groups when a rat did not consume the daily rat chow allotment in entirety.

#### Body weight, food consumption, and caloric intake

Daily measurement of body weight (g) was carried out among the five groups using a covered rodent triple beam balance (Ohaus, Parsippany, NJ). We measured daily consumption of rat chow (g) during baseline and during the 2 week experimental protocol to determine sodium intake as well as caloric intake (in kcal). We also accounted for the food “spilled” into the collection funnel of the cage by measuring food trapped with a paper baffle. Caloric intake from rat chow and either 20% fructose or 20% glucose was calculated from the daily amount of chow and the volume of water consumed (mL). The rat chow had an energy density of 3 kcal/g (Harlan Data Sheet 8640). The energy density of fructose or glucose is 4 kcal/g.

#### Water consumption/urine excretion

The volume of distilled water or water containing 20% fructose or 20% glucose consumed was measured gravimetrically and recorded daily to the nearest milliliter. The volume of urine excreted (see collection methods below) was also collected and measured by weight using an Ohaus electronic balance (Mettler Toledo, Columbus, OH).

#### Sodium excretion and sodium balance

Urine from each individual metabolic cage drained over a mesh wire feces trap and into clean 50 mL conical centrifuge tubes. It was collected in 24 h periods over 19 days; every day at 12:00 h. Metabolic cage pans were replaced with clean bottoms daily to reduce contamination of urine samples from minuscule amounts of food and/or feces. Urine volumes were recorded daily. Urine samples were transferred to Eppendorf tubes, spun at 10K rpm for 5 min to remove particulate contamination and sediment, and placed in a −80 freezer. Daily sodium excretion values were calculated from 24 h urine volumes and sodium concentrations measured by a Nova Biomedical 1 electrolyte analyzer (Waltham, MA). Total cumulative sodium balance was calculated by summing the sequential daily differences between intake and total sodium excretion and expressed in mEq ± SEM over a period of 19 days (Beierwaltes et al. 1982).

#### Fecal sodium excretion

Representative fecal samples were collected from all five groups to determine fecal sodium excretion as a percent of total sodium excretion (urinary and fecal sodium excretion). *n *= 3 for C, F, F + HS, and HS groups, and *n *= 4 for the G + HS group. Individual 24 h fecal samples were transferred to 50 mL conical centrifuge tubes and placed in a −80 freezer until further analysis. Fecal samples were thawed to room temperature, allowed to air dry, and total fecal dry weight was recorded. One gram of feces was combined with 4 mL of distilled water and homogenized with a Tissuemiser (Fisher Scientific, Pittsburgh, PA). The fecal slurry was transferred to a 1.5 mL eppendorf, spun down at 1000 *g* at 4°C for 5 min, the supernatant pipetted into a new eppendorf. Daily fecal sodium excretions were calculated from sodium concentrations using a Nova Biomedical 1 Electrolyte Analyzer (Waltham, MA) and total fecal mass to achieve a 24 hr fecal sodium excretion and fecal excretion as a percent of total sodium excretion (urinary plus fecal sodium excretion) (Beierwaltes et al. 1982).

#### Urinary nitric oxide

At Day 19, the 24 h urine collections were sampled for excretion of nitric oxide (NO) metabolites (NO_2_/NO_3_) as an index of renal NO production. A quantity of 200 *μ*L of Anti‐Anti, an antibiotic and antimycotic, (Life Technologies, Grand Island, NY) was placed in the urine collection tubes prior to collection to prevent growth of bacteria. NO_2_/NO_3_ concentration was measured using a Nitrate/Nitrite Colorimetric Assay Kit (Cayman Chemical, Ann Arbor, MI) via the Griess Reaction. NO_2_/NO_3_ excretion was calculation by multiplying the concentration of NO_2_/NO_3_ by the twenty four hour urine volume. Units are expressed as *μ*mol/24 h.

#### Creatinine clearance

Creatinine was measured in the 24‐h urine samples collected during the last day while the rats were in a metabolic caging, and in the plasma sampled during terminal collection of trunk blood. Blood samples were spun at 255 *g* at 4°C for 10 min, and the plasma was separated and stored at −80°C until creatinine analysis. Creatinine clearance was calculated by multiplying the concentration of urinary creatinine by the 24‐h urine volume, dividing by the plasma creatinine concentration, and then correcting to units of ml/min per gram kidney weight (kw). Kidneys were excised and capsules removed, blotted, and weighed. Clearance values were expressed as mL/min/gkw. (C, *n *= 7, F, *n *= 6, F+HS, *n *= 12, HS, *n *= 8, and G + HS, *n *= 6).

#### Blood glucose and plasma insulin levels

We measured blood plasma glucose and plasma insulin levels at the conclusion of the protocol. Following killing, blood droplets were collected from trunk blood to measure fasting plasma glucose levels. For 12 h prior to killing, the rat groups were fasted, but allowed access to water. Blood glucose was measured with a True Result Blood Glucose Monitor (CVS, Woonsocket, RI). Plasma samples (containing 6% EDTA), stored at −80, were thawed and insulin levels were measured using a rat insulin EIA kit (Cayman Chemical, Ann Arbor, MI) according to the manufacturer's instructions. Samples containing visible amounts of hemolysed blood were excluded from the data set.

### Protocol 2: Fructose and a high salt diet with ad‐libitum sodium intake

This second protocol was run in a similar fashion as protocol 1, but without the limitations of pair‐feeding, as all chow consumption was ad‐libitum. Also, this group did not contain a glucose‐fed group. This was run to assess the effect of fructose and high salt diet on plasma renin activity and total (active + inactive pro‐) renin without the restrictive effects of pair‐feeding (Protocol 1). Rats weighing between 280 and 300 g were immediately placed on either distilled water or drinking water containing 20% fructose. Blood pressures were not measured in this protocol. Rats were allowed free access to food and water. The four groups included: a control group (C); given distilled water and normal rat chow (*n *= 7), a high salt group (HS); given distilled water and high salt (4% NaCl) rat chow over the second week of the protocol (*n *= 7), a 20% fructose group (F); given 20% fructose in drinking water and normal rat chow (*n *= 7) throughout the protocol, and a 20% fructose and high salt group (F+HS), given 20% fructose in the drinking water throughout the protocol and high salt chow over the second week of the protocol. (*n *= 7).

#### Plasma renin activity and total renin

At the conclusion of the protocol, rats were killed via decapitation using a rat guillotine (Harvard Bioscience, Cambridge, MA). Promptly after decapitation, the first 3 sec of trunk blood were collected for PRA analyses in 15 mL tubes containing 200 *μ*L of 6% EDTA in 0.9% NaCl. This collection method has been approved by our IACUC as it avoids the abnormal stimulation of PRA by the renal baroreceptor reflex and by anesthesia, which would otherwise increase PRA (Pettinger 1978). Additional blood samples were taken using either 200 *μ*L of 6% EDTA (Sigma‐Aldrich, St. Louis, MO), or 100 *μ*L of sodium heparin (Sagent Pharmaceuticals, Schaumberg, IL) as anticoagulants. Blood samples were spun at 1000*g* at 4°C for 10 min, and the plasma was separated and stored at −80°C until further analysis. PRA was analyzed by generation of ANG I (ng ANG I·mL^−1^ h^−1^ min^−1^) using a Gamma Coat RIA kit (DiaSorin, Stillwater, MN) as previously described, and according to the manufacturer's instructions. Following blood collection, the gross physical appearance of the kidneys was screened for abnormalities, and none were found. Kidneys were excised, decapsulated, blotted, and weighed. Additional blood samples were collected using either 200 *μ*L of 6% EDTA (Sigma‐Aldrich) or 100 *μ*L of sodium heparin (Sagent Pharmaceuticals, Schaumberg, IL) as anticoagulants. Blood samples were spun at 1000 *g* at 4°C for 10 min, and the plasma was separated and stored at −80°C until further analysis. PRA was analyzed by generation of ANG I (ng ANG I mL^−1^ h^−1^) using a Gamma Coat RIA kit (DiaSorin, Stillwater, MN) as previously described (Macgriff et al. 2014) and according to the manufacturer's instructions. Following blood collection, the kidneys were excised, decapsulated, blotted, and weighed.

To determine if the different diets had any effect on changes in inactive renin (prorenin) in fructose‐fed and high salt‐fed rats, we measured total renin (active and inactive prorenin). Total renin was measured in rat plasma in the four groups using an enzyme‐linked immunosorbent assay (ELISA), which identifies an epitope present on both the active and prorenin molecule (Molecular Innovations, Novi, MI, USA), The assay was run according to the manufacturer's instructions, as we have previously described (Macgriff et al. 2014).

#### Body weight, food and water consumption

Daily measurements of body weight (g) were carried out among the five groups using a rodent covered triple beam balance (Ohaus, Parsippany, NJ). Both food and water consumption were also measured daily (as described above).

#### Plasma blood glucose, plasma insulin, and serum lipid panel

We measured plasma glucose and insulin levels at the conclusion of the 2‐week protocol. Following killing, fasting blood glucose levels were determined using a True Result Blood Glucose Monitor (CVS, Woonsocket, RI). Plasma samples collected into 6% EDTA samples were measured for plasma insulin levels using a rat insulin EIA kit (Cayman Chemical, Ann Arbor, MI), according to the manufacturer's instructions.

To detect changes in cholesterol and triglycerides with our diet regimens, we performed a lipid panel using rat serum on Siemens healthcare diagnostic analyzer (Siemens, Malvern, PA) according to the manufacturer's instructions. This assay provides total serum cholesterol, triglycerides, low‐density and high‐density lipoproteins.

### Protocol 3: glucose tolerance test

To determine whether fructose‐induced, salt‐sensitive hypertension preceded insulin resistance, we performed glucose tolerance tests (GTT) in a separate protocol. We used groups of rats including: a control group (C) fed distilled water and normal rat chow (*n *= 4), a 20% fructose group (F) fed 20% fructose in the drinking water for 2 weeks plus normal rat chow (*n *= 5), and a 20% fructose plus high salt (4%) group (F + HS), fed 20% fructose in drinking water for 2 weeks, and 4% high salt rat chow fed during the final 7 days (*n *= 3). These diet protocols were designed to be similar to our previous protocols.

At the conclusion of the 2‐week dietary regimens, male Sprague–Dawley rats (Charles River, Wilmington MA) weighing 300–500 g were fasted overnight but allowed free access to (distilled) drinking water. On the day of the experiment, rats were anesthetized via intraperitoneal injection with thiobutabarbital, 125 mg/kg b.w. (Inactin, Sigma Aldrich, St. Louis, MO), and placed on a heated surgical table to maintain constant body temperature (BrainTree Scientific, Braintree, MA). A tracheotomy was performed using PE‐240 tubing to allow free breathing of room air. A femoral cut down was performed to cannulate the femoral artery with PE‐50 catheters. Arterial blood was collected and plasma glucose measured with a True Result Blood Glucose Monitor (CVS, Woonsocket, RI). Blood glucose was measured at baseline and following an intragastric bolus of glucose by gavage (3 mg/kg bw in 0.5 mL water) at sequential timepoints. Blood was sampled before and 5, 10, 15, 30, 60, 120 min after administering the gavage.

## Results

### Protocol 1: Blood pressure and cumulative sodium balance in response to fructose and a high salt diet with paired‐feeding to control sodium intake

**Figure 1:**
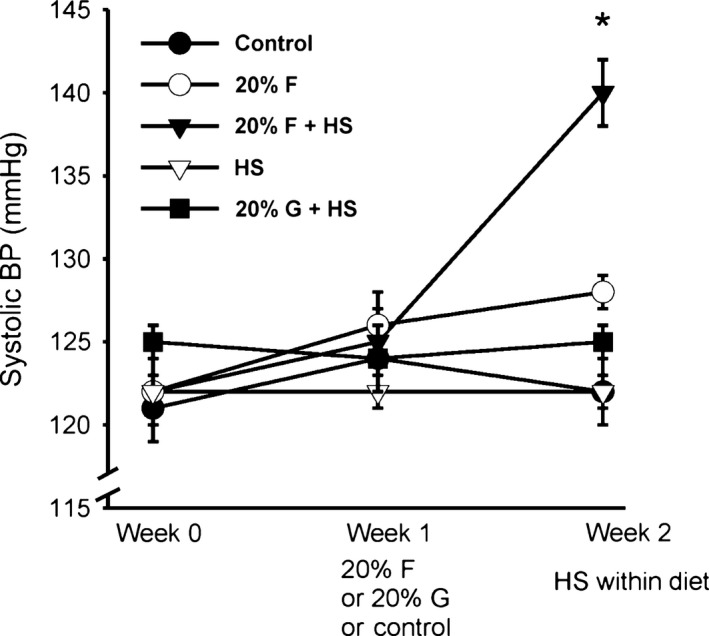
Systolic blood pressure over the study period. With the addition of high salt chow in week 2, systolic blood pressure in the 20% fructose plus high salt group increased significantly. However, blood pressure was unchanged in all other groups, including both the high salt control and 20% glucose plus high salt groups. (*P < 0.05 vs. previous period).

In the first protocol (sodium balance; pair feeding), we employed five different dietary groups of rats over 2 weeks of treatment after obtaining baseline values. These included group (1) control (C); given distilled water and fed a normal rat chow (*n *= 9); group (2) given 20% fructose (F) in the drinking water and fed normal rat chow (*n *= 9); group (3) given 20% fructose in the drinking water and fed high salt (F + HS) rat chow (4% NaCl) during the second week (*n *= 18); group (4) given distilled water and fed high salt (HS) rat chow during the second week (*n *= 9), and group (5) given 20% glucose and fed high salt (G + HS) rat chow during the second week (*n *= 8). In this first protocol, the groups were pair‐fed so that the amount of chow (and salt) from the chow remained constant across groups.

systolic blood pressure in the five groups at baseline were: C) 121 ± 2, F) 122 ± 2, F + HS) 122 ± 1, HS) 122 ± 1, and G+HS) 125 ± 1 mmHg (Fig. 1). There were no differences between groups in systolic blood pressure at baseline. Initiation of experimental diets: 20% fructose (F and F + HS) and 20% glucose (G + HS) started after baseline measurements were obtained. At the end of the first week of experimental diets, systolic blood pressure remained unchanged in all five groups: C) 124 ± 3, F) 126 ± 2, F+HS) 125 ± 1, HS) 122 ± 1, and G + HS) 124 ± 2 mmHg. At the beginning of the second week of experimental diets, normal chow was replaced with a 4% high salt diet in the F + HS, HS, and G + HS groups. Systolic blood pressure by the end of week 2 were: C) 122 ± 2, F) 128 ± 1, F+HS) 140 ± 2, HS) 122 ± 1, and G + HS 125 ± 1. Blood pressure significantly increased only in the 20% fructose plus high salt group (125 ± 1 to 140 ± 2 mmHg, *P *< 0.01) while systolic pressure the HS and G + HS groups remained unchanged (Fig. 1) in response to the addition of high salt to the diet. Overall, 20% fructose in the presence of high salt increased blood pressure while fructose alone, glucose or high salt alone had no effect on systolic blood pressure during the protocol.

#### Body weight

**Figure 2:**
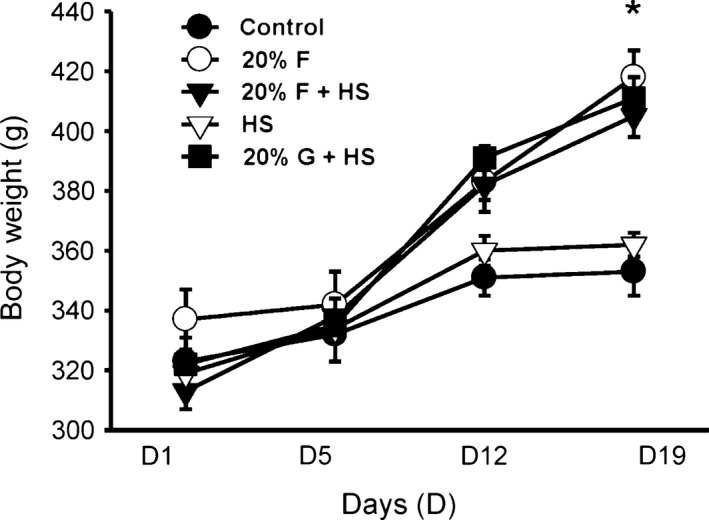
Body weight over the study period. Body weight significantly increased in the groups receiving 20% fructose or 20% glucose in their water. Since chow was pair‐fed, by the end of the study period, there were significantly higher body weights in the fructose‐ or glucose‐supplemented groups. (*P < 0.05 vs. control).

In this first protocol, pair feeding was carried out to ensure that the five groups of rats had equivalent solid food and sodium intake. There were no significant differences in body weight between the five groups at the beginning of the experiment (Table 1). By the end of the protocol, as expected, all five groups significantly increased in body weight. Furthermore, there were differences in body weight between C and HS groups compared to the three groups (F, F + HS and G + HS) which were heavier (*P *< 0.05) due to receiving additional calories from fructose/glucose in their drinking water. However, there were no differences in body weight between the 3 groups receiving either 20% fructose or glucose in their water (Fig. 2).

**Table 1:** Body weight (g), protocol 1

	C	F	F + HS	HS	G + HS
Start baseline	323 ± 8	337 ± 10	331 ± 5	319 ± 5	322 ± 2
End baseline	332 ± 9	341 ± 11	338 ± 6	334 ± 4	335 ± 3
End week 1	351 ± 6	383 ± 10	383 ± 5	359 ± 5	391 ± 4
End week 2	353 ± 8	418 ± 9	405 ± 7	362 ± 4	411 ± 7
Δ BW	30 ± 1^a^	81 ± 10 a, b	74 ± 3 a, b	43 ± 3^a^	89 ± 7 a, b

Rat body weights over the course of the protocol (g).

C, control diet group; F, 20% fructose fed group; F + HS, 20% fructose fed plus high salt (4.0%) chow; HS, rats fed the high salt rat chow; G + HS, 20% glucose fed plus high salt (4.0%) chow.

a *P* < 0.05 versus starting weight.

b *P* < 0.05 versus non‐sugar supplemented rats.

#### Food intake

There were no differences in chow consumption across all five groups during the baseline period (Table 2A). However, with the addition of 20% fructose (F and F + HS groups) or the G + HS group, food consumption decreased on average by 18% (*P *< 0.001). Food consumption *Table 2A) and caloric consumption (Table 2B) further decreased on average 27% with the addition of high salt chow during week 2 (*P *< 0.0001). Due to the pair‐feeding protocol, caloric intake in groups lacking 20% fructose or 20% glucose explain the differences in final body weight (Table 1). After the baseline period, the F, F + HS and G + HS groups received significantly more calories due to fructose/glucose supplemented water (Table 2C) than non‐supplemented groups C and HS (*P *< 0.0001), as chow intake was controlled by the pair feeding.

**Table 2:** Chow intake, Protocol 1

	Control	F	F + HS	HS	G + HS
(A) Daily rat chow intake (g)					
Baseline	20.3 ± 0.4	20.2 ± 0.4	20.2 ± 0.3	19.6 ± 0.5	19.8 ± 0.5
Week 1	16.7 ± 0.4	15.7 ± 0.4	16.2 ± 0.3	17.3 ± 0.2	17.3 ± 0.2
Week 2	12.8 ± 0.4	12.5 ± 0.4	10.5 ± 0.4	11.1 ± 0.1	11.1 ± 0.1
(B) Daily caloric intake (kcal) from rat chow					
Baseline	61.0 ± 1.3	60.1 ± 1.1	60.7 ± 0.8	58.9 ± 1.6	59.4 ± 1.6
Week 1	50.2 ± 1.3	47.0 ± 1.3	48.6 ± 0.8	52.0 ± 0.5	51.0±0.8
Week 2	38.3 ± 1.3	37.5 ± 1.1	31.5 ± 1.2	33.2 ± 0.2	29.4 ± 1.9
(C) Additional calories from added sugar					
Baseline	0	0	0	0	0
Week 1	0	32.8 ± 3.2	32.8 ± 1.6	0	40.0 ± 2.4
Week 2	0	36.0 ± 3.2	43.2 ± 2.4	0	48.0 ± 4.0
(D) Daily sodium intake (mEq)					
Baseline	3.46 ± 0.07	3.44 ± 0.06	3.44 ± 0.04	3.34 ± 0.09	3.37 ± 0.09
Week 1	2.84 ± 0.07	2.66 ± 0.07	2.75 ± 0.04	2.95 ± 0.03	2.89 ± 0.04
Week 2	2.17 ± 0.07	2.12 ± 0.06	17.86 ± 0.67	18.80 ± 0.13	16.65 ± 1.07

C, control diet group; F, 20% fructose fed group; F + HS* *= 20% fructose fed plus high salt (4.0%) chow; HS, rats fed the high salt rat chow; G + HS, 20% glucose fed plus high salt (4.0%) chow.

#### Sodium intake

During the baseline period, there were no differences in sodium intake across all five groups (Table 2D). When the fructose/glucose protocol was initiated, chow intake decreased (Table 2D) due to the addition of calories obtained from 20% fructose or 20% glucose, so that sodium intake was decreased in all five groups due to pair‐feeding. During the final week with the addition of high salt diet to groups F + HS, HS, and G + HS, sodium intake significantly increased by nearly sixfold (*P *< 0.001).

#### Water consumption/urine excretion

**Figure 3:**
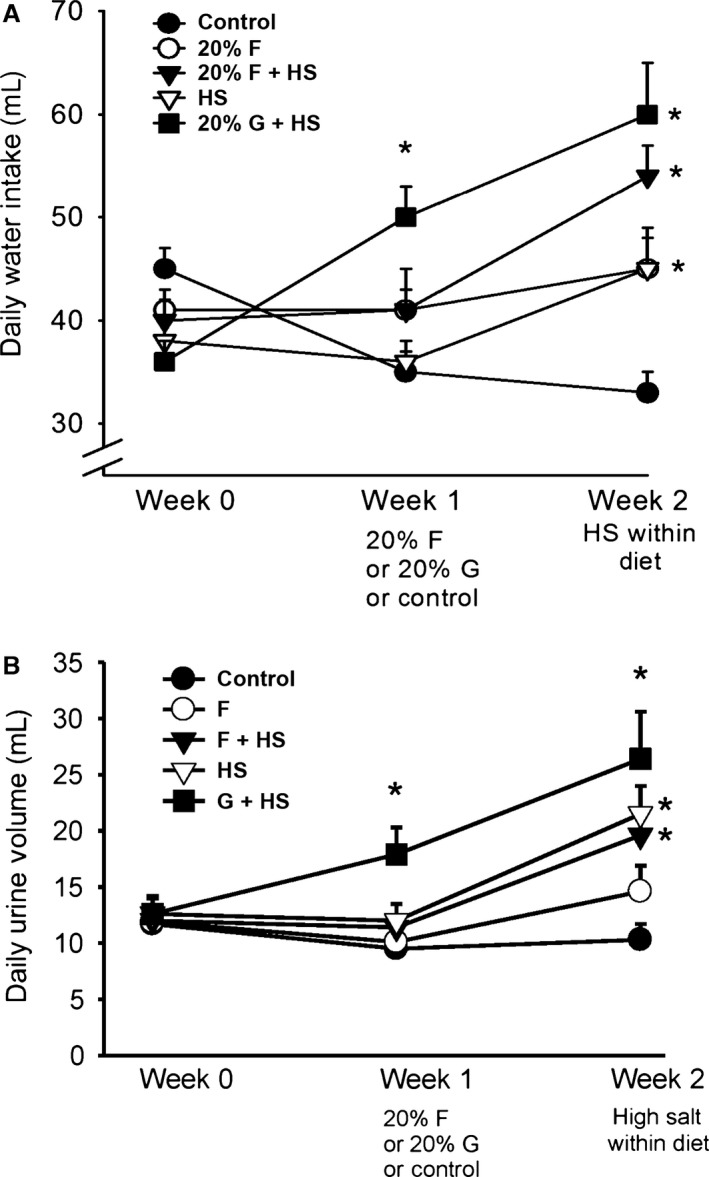
Water balance. Water intake (A, upper panel) increased only in the 20% glucose plus high salt diet during the first week. However, water intake increased during the second week in all three groups receiving a high salt diet. (*P < 0.05 consumption vs. the previous week). Daily urine volume (B, lower panel) increased as the volume consumed increased. Urine volume increased significantly in the 20% glucose plus high salt group in the first week. Urine volumes increased in all three high salt‐treated groups during the second week. (*P < 0.05, as measured within same group from previous week).

During baseline, there were no differences in the amount of water consumed across the five groups (Fig. 3A). In the first week of the fructose/glucose protocol, water consumption in the C group decreased (Table 3A). In F, F + HS, and HS groups, water consumption remained constant. However, the G + HS group increased water consumption by 39% (*P *< 0.0001) with the addition of 20% glucose to the water. During the second week (with salt), water consumption in the C group was unchanged. Water consumption increased in the F group 2 by 10% (*P *< 0.01). As expected with the addition of the high salt diet to F + HS* *< HS and G + HS during week 2, (Table 3A) water consumption increased.

**Table 3:** Daily water balance, Protocol 1

	Control	F	F + HS	HS	G + HS
(A) Daily water intake (mL)					
Baseline	45 ± 2	41 ± 2	40 ± 2	38 ± 2	36 ± 2
Week 1	35 ± 2	41 ± 4	41 ± 2	36 ± 2	50 ± 3
% change	−22	0	3	−5	39
*P*‐value	0.003	n.s.	n.s.	n.s.	0.0001
Week 2	33 ± 2	45 ± 4	54 ± 3	45 ± 3	60 ± 5
% change	−6	10	32	25	54
*P*‐value	n.s.	0.01	0.0001	0.0006	0.01
(B) Daily urine volumes (mL)					
Baseline	11.7 ± 1.4	11.9 ± 1.3	12.0 ± 1.3	12.6 ± 1.6	12.6 ± 1.4
Week 1	9.5 ± 1.3	10.1 ± 1.9	11.4 ± 1.0	12.0 ± 1.5	17.9 ± 2.4 *
Week 2	10.3 ± 1.4	14.6 ± 2.3	19.6 ± 1.6*	21.5 ± 2.5*	26.4 ± 4.2*

C, control diet group; F, 20% fructose fed group; F + HS, 20% fructose fed plus high salt (4.0%) chow; HS, rats fed the high salt rat chow; G + HS, 20% glucose fed plus high salt (4.0%) chow. *P < 0.05 versus the previous week.

There were no differences in urine volumes during baseline (Table 3B). In the first week of the fructose/glucose protocol, urine volumes were unchanged except for the G + HS group, where urine volume increased in response to increased consumption of the water containing 20% glucose (see above and Fig. 3B). As expected, during the second week with the replacement of regular rat chow with high salt chow, urine volume increased significantly (Table 3B) in F + HS, HS, and G + HS groups.

#### Sodium excretion and cumulative sodium balance

To test whether fructose increased sodium retention, we measured daily sodium excretion. During baseline, urinary sodium excretion among the five groups was consistent across groups, though the G + HS had a somewhat higher sodium excretion (*P *< 0.04) (Table 4).

**Table 4:** Sodium excretion (mMoles/24 h), Protocol 1

	Control	F	F + HS	HS	G + HS
Baseline	1.35 ± 0.10	1.56 ± 0.08	1.50 ± 0.07	1.72 ± 0.18	1.83 ± 0.14
Week 1	1.31 ± 0.01	1.11 ± 0.13	1.25 ± 0.08	1.73 ± 0.10	1.77 ± 0.13
% change	−3	−29	−17	<1	−3
*P* ‐value	n.s.	0.02	0.001	n.s.	n.s.
Week 2	1.13 ± 0.07	1.20 ± 0.10	5.33 ± 0.21	7.14 ± 0.33	6.12 ± 0.77
% change	−14	8	326	313	246
*P* ‐value	0.01	n.s.	0.0001	0.0001	0.0001

Protocol 1:Urinary Sodium Excretion (mMol/24 h).

C, control diet group; F, 20% fructose fed group; F + HS, 20% fructose fed plus high salt (4.0%) chow; HS, rats fed the high salt rat chow; G + HS, 20% glucose fed plus high salt (4.0%) chow.

**Figure 4:**
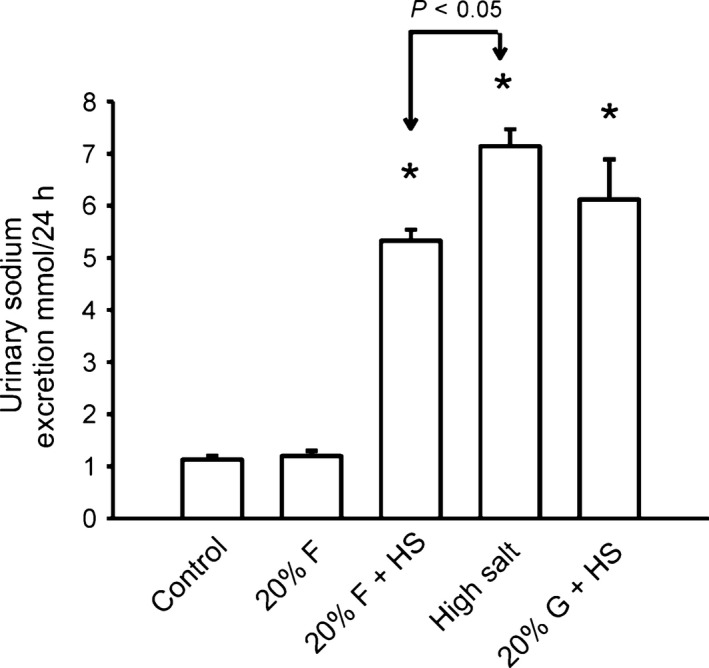
Sodium Excretion Sodium excretion is presented from week 2, with the introduction of the high salt chow. Sodium excretion was increased in all three groups fed high salt chow. (*P < 0.05) compared to control, or non‐high salt treated groups. However, this increased sodium excretion was blunted in 20% fructose plus high salt group.

After introduction of experimental diets, urinary sodium excretion was unchanged in controls as well as in the G + HS group. We found that sodium excretion was decreased in both the fructose‐treated groups, groups F and F + HS. With the introduction of high salt diet in the second week, sodium excretion increased in groups F + HS, HS, and G + HS (Table 4). However, sodium excretion was less in the F + HS group compared to its appropriate HS control (Fig. 4), but sodium excretion was not decreased in G + HS compared to the HS group, suggesting increased sodium retention in the presence of fructose and high salt.

**Figure 5:**
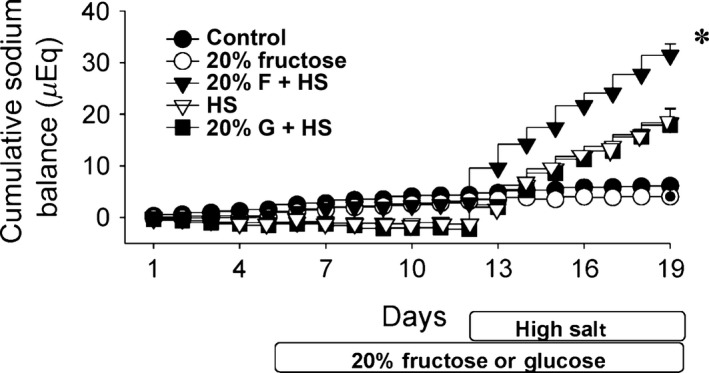
Cumulative sodium balance. There are no differences among groups in cumulative sodium balance prior to the introduction of high salt diet. Cumulative sodium balance increased during the final week in all three groups fed high salt diet. The 20% fructose + high salt group retained the most sodium; significantly more than HS or G + HS groups (*P < 0.008).

To examine if fructose‐fed rats were retaining sodium, we measured cumulative sodium balance over course of the protocol. Cumulative sodium balance takes into account the progression of both sodium intake and excretion (Fig. 5), and thus net retention over time. There were no differences in cumulative sodium balance between the five groups during baseline or during the first week of the protocol with or without 20% fructose or glucose. However, in the final week with the replacement of normal rat chow to high salt rat chow, (groups F + HS, HS and G + HS), we found significant increases in cumulative sodium balance in all three groups given high salt diet compared to normal salt controls (Fig. 5). Cumulative sodium balance in the five groups at the end of the protocol (Day 19) were: C) 6.12 ± 1.15, F) 4.04 ± 1.90, F + HS), 31.43 ± 2.21 (*P *< 0.0004), HS) 18.35 ± 2.67 (*P *< 0.003), G+HS) 17.90 ± 3.26 (*P *< 0.01) mEq sodium (*P*‐values vs. control, adjusted for multiple comparisons in ANOVA). The sodium retained in F + HS group was almost double that of either HS or G + HS groups (*P *< 0.008), corresponding to the significantly decreased sodium excretion (Fig. 4) in the F+HS group and concurrent with the period in which blood pressure increased in the F + HS group (Fig. 1). These data suggest fructose (but not glucose)‐induced increased sodium retention resulting in salt‐sensitive increased blood pressure.

#### Fecal sodium excretion

To determine if fructose‐impaired sodium absorption occurs in the intestines, we measured whether fecal sodium excretion would increase in response to the high salt diet. We measured fecal sodium excretion over the course of the protocol in a subset of rats within each group (Table 5). During baseline, there were no differences in fecal sodium excretions. Addition of 20% fructose and 20% glucose during the initial week of the fructose/glucose protocol also had no effect on fecal sodium excretion. When high salt was added to the diets, fecal sodium excretion did not increase (Table 5). If fecal sodium excretion was calculated as a percent of total sodium excretion, it was 2–5% of total sodium excretion during normal diet and decreased significantly with the addition of high salt diet to <1%. This suggests that the kidney is responsible for excreting a majority of consumed sodium regardless of the amount. During all dietary permutations, sodium reabsorption in the intestines was remarkably consistent.

**Table 5:** Fecal sodium excretion mMol/24 h and expressed as % total Sodium excretion: Protocol 1

Group	Baseline	Week 1	Week 2
Control	0.05 ± 0.01	0.04 ± 0.01	0.02 ± 0.01
% total	3.8 ± 0.4	3.9 ± 0.2	2.5 ± 0.3 %
Fructose	0.05 ± 0.01	0.04 ± 0.01	0.03 ± 0.01
% total	3.3 ± 0.5	4.2 ± 0.4	2.7 ± 0.2 %
Fructose + high salt	0.05 ± 0.01	0.04 ± 0.01	0.02 ± 0.01
% total	3.9 ± 0.4	3.2 ± 0.2	0.4 ± 0.1 %
High salt	0.05 ± 0.01	0.04 ± 0.01	0.03 ± 0.01
% total	4.0 ± 0.4	3.1 ± 0.4	0.5 ± 0.1 %
Glucose + high Salt	0.05 ± 0.01	0.03 ± 0.01	0.02 ± 0.01
% total	3.6 ± 0.6	2.4 ± 0.2	0.5 ± 0.1 %

Values are expressed in mMol/24 h, and also as a % of the total sodium excretion (urinary plus fecal). Note high salt was added to certain diets only during week 2.

#### Urinary nitric oxide excretion

**Figure 6:**
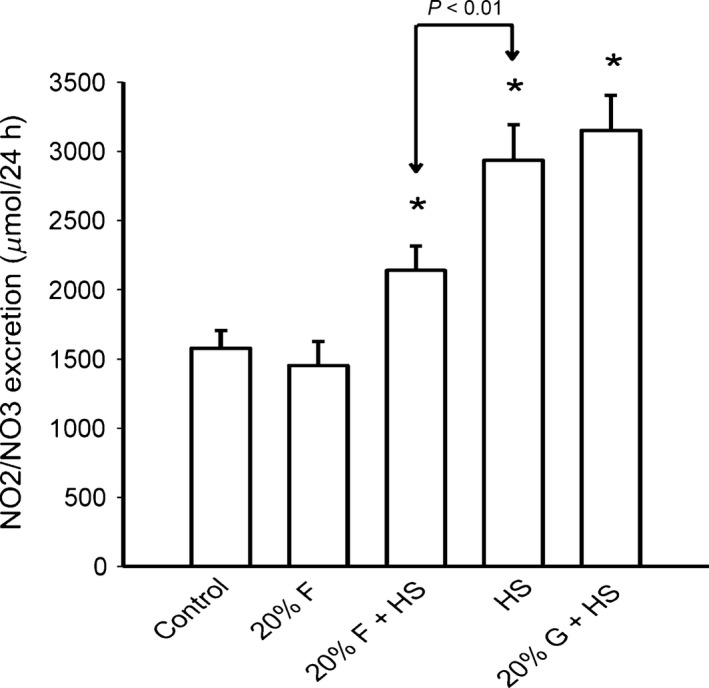
NO_2_/NO_3_ excretion: NO_2_/NO_3_ excretion was higher than normal salt controls in all three groups fed a high salt chow (*P < 0.05), but the rise in NO_2_/NO_3_ excretion was blunted in the 20% fructose + high salt group compared to either high salt or 20% glucose + high salt groups (*P *< 0.01).

To test if 20% fructose and/or a high salt diet influenced changes urinary excretion of nitric oxide metabolites (NO_2_/NO_3_), we sampled NO metabolic excretion across all five groups (Fig. 6). NO_2_/NO_3_ excretion as a biomarker for renal NO in the five groups were: C) 1579 ± 126, F) 1452 ± 175 F + HS) 2139 ± 178, HS) 2935 ± 256, and G + HS) 3152 ± 254 *μ*mol/24 h. Collectively, the high salt groups (F + HS, HS, and G + HS) had significantly higher NO_2_/NO_3_ excretion than groups not given high salt (C and F). NO_2_/NO_3_ excretion in the F + HS group was significantly less than either the HS or the G+HS groups (Fig. 6), suggesting fructose in the presence of high salt impairs NO_2_/NO_3_ excretion or decreases NO bioavailability.

#### Creatinine clearance

At the end of the protocol, there were no differences in creatinine clearance in the five groups: C) 0.84 ± 0.14, F) 0.88 ± 0.16, F+HS) 0.90 ± 0.04, HS) 0.94 ± 0.05, or G+HS) 1.16 ± 0.15 mL/min/g kidney weight, suggesting glomerular filtration rate remained autoregulated.

#### Blood glucose and plasma insulin

**Figure 7:**
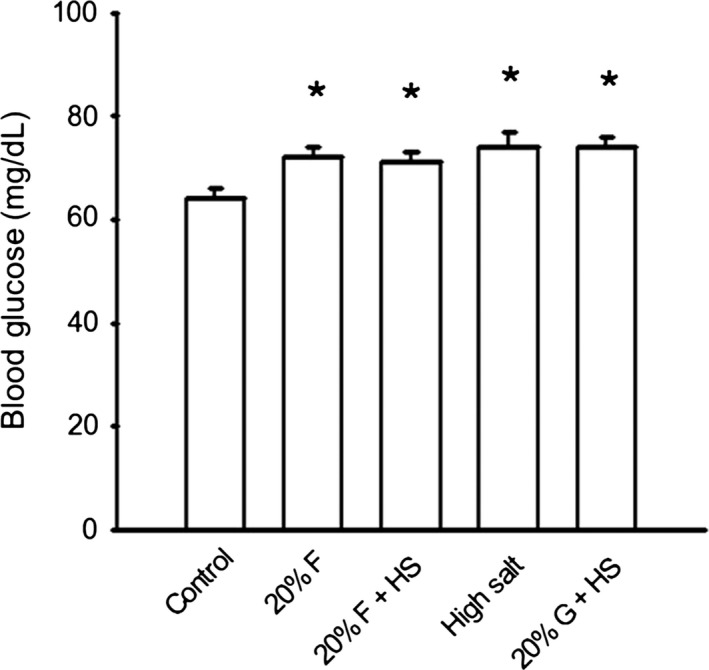
Blood glucose. Fasting blood glucose was slightly but significantly (*P *< 0.05) elevated in all groups versus control. However, despite these increased, all groups remained normoglycemic

Glucose was measured in the blood of all rats at the conclusion of the study to evaluate if the onset of hypertension preceded indicators the development of pre‐diabetes. Fasting blood glucose levels in the five groups were: C) 64 ± 2, F) 72 ± 2, F+HS) 71 ± 2, HS) 74 ± 3, and G+HS) 74 ± 2 mg/dL. All four experimental groups had elevated blood glucose (Fig. 7) compared to controls. The fasted rats still had access to their respective experimental water supply. Although there were minor elevations in blood glucose levels in the fructose and glucose‐fed rats, all five groups had blood glucose levels in normal physiological (normoglycemic) ranges.

We also measured plasma insulin in the five groups of rats at the conclusion of the protocol. Insulin levels in the five groups were: C) 1.09 ± 0.18, F) 2.29 ± 0.27, F+HS) 1.94 ± 0.19, HS) 3.19 ± 0.67, and G + HS) 1.75 ± 0.33 ng/mL. Insulin levels were higher in the F, F + HS, and HS groups as compared to control (all *P*‐values less than *P *< 0.05).

### Protocol 2: Fructose and a high salt diet with ad‐libitum sodium intake

This second protocol was run in a similar fashion as protocol 1, but without the limitations of pair‐feeding, as all chow consumption was ad‐libitum. Also, this protocol did not contain a glucose‐fed group.

#### PRA and total renin

**Figure 8:**
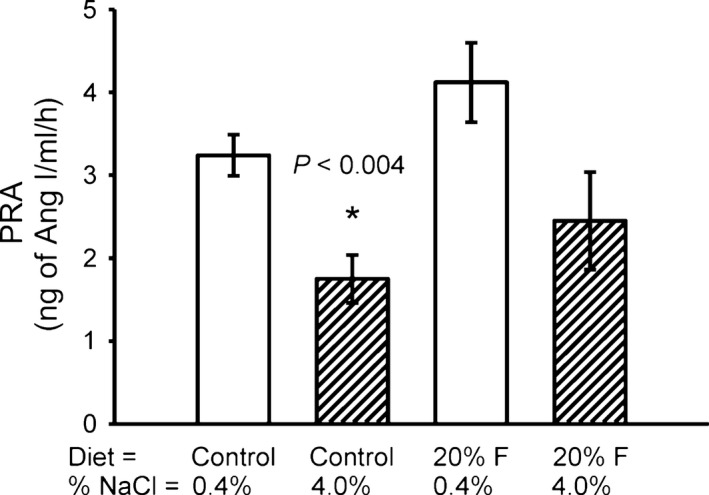
Plasma Renin Activity (PRA). High Salt diet suppressed PRA by 47% (*P *< 0.004). However, in the 20% Fructose + high salt group, the salt suppression of PRA was blunted such that it was not different from normal salt controls.

The values for PRA in our four non‐pair‐fed groups were: C) 3.24 ± 0.27, HS) 1.75 ± 0.31, F) 4.12 ± 0.52, and F + HS) 2.45 ± 0.59 ng Ang I per mL/h. (Fig. 8). As expected, in the HS group, PRA was significantly suppressed as compared to control (*P *< 0.004). However, in the F + HS group, the high salt diet did significantly suppress PRA.

To determine if diet had any effect on changes in inactive renin (prorenin) in fructose‐fed and high salt‐fed rats, we measured total renin (an assay for both active renin and inactive prorenin). Total renin in the four groups were: C) 18.0 ± 1.9, HS) 11.4 ± 2.7 F) 19.1 ± 1.4, and F+HS) 15.5 ± 2.3 ng/mL. Total renin levels followed a similar profile to PRA suggesting no unusual dissociation of active and total renin.

#### Blood glucose, insulin levels, and lipid panel

At the end of the protocol, blood glucose in the four groups were: C) 80 ± 3, HS) 75 ± 3, F) 88 ± 4, and F + HS) 80 ± 3. The F group had significantly higher blood glucose compared to HS (*P *< 0.01) but not compared to C. However, the F + HS group did not have higher blood glucose than the other groups. Overall, blood glucose levels are within normal physiological range.

Serum insulin in the four groups was: C) 2.3 ± 0.31. HS) 3.66 ± 0.64, F) 5.55 ± 1.25, and F+HS) 1.83 ± 0.19 ng/mL. We found elevated insulin levels only in the F group (*P *< 0.04).

**Figure 9:**
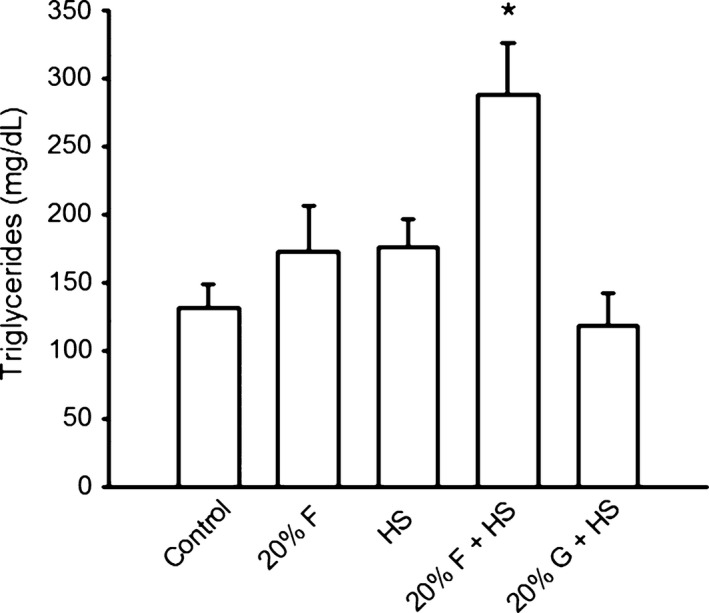
Triglycerides Plasma triglycerides were increased (*P *< 0.05) only in the fructose + high salt group by the end of the study period.

We performed a complete lipid panel on the four groups of rats (Table 6). Overall, there were no changes in total cholesterol. However, the F+HS group had significantly elevated triglycerides compared to controls (Fig. 9). We also saw a significant decrease in the HDL levels in the F + HS group compared to controls.

**Table 6:** Lipid Panel (mg/dl): Protocol 2

	C	F	HS	F + HS
Cholesterol	67 ± 4	61 ± 4	72 ± 4	61 ± 5
Triglycerides	132 ± 17	173 ± 34	176 ± 21	288 ± 38^a^
LDL	11 ± 1	8 ± 1	12 ± 1	7 ± 1
HDL	52 ± 3	45 ± 3	51 ± 4	35 ± 5^a^

Protocol 2: Lipid Panel.

20% F, 20% fructose fed group; 20% F + HS, 20% fructose fed plus high salt (4.0%) chow.

a *P* < 0.05 versus control.

#### Body weight, food and water consumption

We measured body weight in the four groups of rats. At the beginning of the experiment, there were no differences in body weight among the four groups. As expected, over 2 weeks all four groups significantly increase in body weight (Table 7A). By the end of 2 weeks without pair‐feeding, regardless of the diet, there were no differences in body weight among the four groups.

**Table 7:** Body weight, food and water intake, Protocol 2

Body Weight (g)	C	HS	F	F + HS
(A) Body weight (g)				
Start	302 ± 4	297 ± 14	284 ± 5	282 ± 5
End	409 ± 6	392 ± 7	393 ± 11	374 ± 14
(B) Food (g)				
Week 1	29.9 ± 0.3	30.4 ± 0.6	23.3 ± 0.8	23.4 ± 0.9
Week 2	29.3 ± 1.1	26.8 ± 0.6	21.5 ± 0.9	15.4 ± 1.2
(C) Water intake (mL)				
Week 1	44 ± 2	43 ± 2	49 ± 3	50 ± 3
Week 2	44 ± 3	69 ± 2 a	57 ± 2 b	70 ± 4 1

a *P* < 0.001 versus week 1.

b *P* < 0.003 versus control.

Daily food intake was measured in the four groups over 2 weeks (Table 7B). Food consumption remained constant in the control group over 2 weeks. Rats consuming 20% fructose (F and F + HS) consumed less food than the control group. Food consumption decreased in the HS and also in F + HS groups with the introduction of high salt food to the diet during the second week.

We measured daily water intake in the four groups of rats (Table 7C). During the first week, there were no significant differences in water intake in the four groups. During week 2 with the introduction of high salt diet to the HS and F + HS groups, water intake increased. In week 2, water intake was also higher in the F group compared to control.

#### Protocol 3: glucose tolerance test

**Figure 10:**
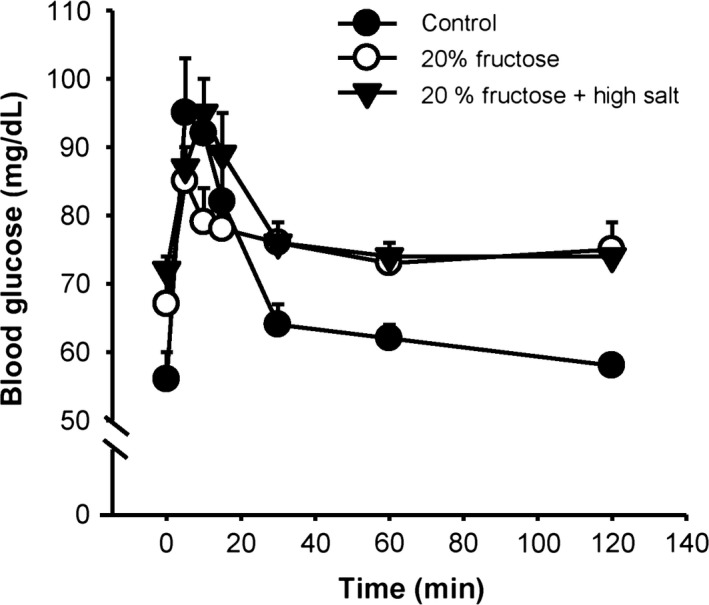
Glucose tolerance test: Fructose‐fed rats had a higher baseline blood glucose level, but all three groups increased to a similar blood glucose level in a similar time course in response to the bolus of glucose. All groups returned to their respective baselines in a similar fashion.

We wanted to test if 2 weeks of either 20% fructose‐feeding or 20% fructose and high salt would result in insulin resistance. Glucose tolerance tests were performed in three groups of rats: 1) control diet (C), 2) 20% fructose (F), and 3) 20% fructose and high salt (F + HS) (Fig. 10). We found (as previously) that the two fructose‐fed groups had a higher baseline blood glucose levels, and all three groups increased blood glucose to a similar levels in response to a glucose bolus. The curve of the results for fructose‐fed or fructose plus high salt was not shifted to the right, and in each case each blood glucose response returned to its original baseline over a similar time course. Thus, blood glucose levels did not exceed 120 mg/dL, nor was there any rightward shift of the blood glucose response curve.

## Discussion

In this study, we found that: (1) moderate fructose intake (20% fructose in drinking water) led to salt‐sensitive hypertension, (2) salt‐sensitive hypertension was induced by fructose but not observed with similar amounts of glucose, (3) the rapid increase in blood pressure was associated with reduced cumulative sodium excretion, positive salt retention, and impaired renal nitric oxide excretion.

In humans, chronically elevated fructose intake is increasingly recognized as a dietary factor linked to hypertension and independently associates with higher blood pressure in adults (Jalal et al. 2010). Beyond just mere correlation, Brown et al. (2008), showed that ingestion of fructose can acutely increase blood pressure. Increases in blood pressure were absent in glucose controls. A positive correlation has also been shown between hypertension and dietary added sugar and salt consumption (Brown et al. 2011). However, it is not clear whether fructose or other added sugars in the diet directly affects renal salt handling during high salt intake in humans. Few patient studies have specifically examined this, and to the best of our knowledge, there have been no interventional trials eliminating both fructose and salt intake from patient diet. In normal rats, blood pressure was unchanged (Johnson et al. 1993) by sucrose on a normal salt diet; however, blood pressure increased in rats on high salt diet receiving sucrose. A recent study also showed that sodium restriction in rats fed very high dietary fructose reduced renal inflammation and oxidative stress (Oudot et al. 2013). Overall, most data suggest that in animal models, combining a high salt diet with enhanced fructose can have deleterious effects on blood pressure control. However, it is unclear whether these effects are due to enhanced renal Na retention, alterations in how salt affects the renin angiotensin system or renal NO production.

In rodents, excessive amounts of fructose (40–60% of caloric intake) in the diet induces hypertension (Hwang et al. 1987; Tran et al. 2009). It has also been shown that these excessive amounts of fructose (40–60%) also induce hyperinsulinemia and hypertriglyceridemia, renal hypertrophy, afferent arteriolar thickening, glomerular hypertension, and vasoconstriction (Sánchez‐Lozada et al. 2007). However, such high experimental doses of fructose do not adequately reflect the amount of added sugar intake within the American diet. Most data indicate that fructose intake represents 20–25% of caloric intake in the highest consumers of added sugars in the US population (Mattson and Higgins 1996). In this study, we provided rats with 20% fructose or glucose in drinking water and found that when high salt diet (4% NaCl) was added, blood pressure rapidly increased about 15 mmHg. During this limited 2 week time course, blood pressure was unchanged by diets containing 20% fructose with normal salt, high salt alone, or in 20% glucose + high salt groups. In agreement with these data, we recently showed that feeding normal Sprague–Dawley rats 20% fructose (in the drinking water) induced salt sensitive increases in blood pressure (Cabral et al. 2014). Previous reports are also in agreement with those data. For example, Reed et al. (1994) reported that fructose (10–20% fructose) induced salt‐sensitive hypertension in Wistar rats (Reed et al. 1994). Thus, our data are consistent with evidence indicating that fructose, but not glucose, induces salt‐dependent increases in blood pressure that occur within 2 weeks of treatment.

The mechanisms by which fructose induces salt‐dependent increase in BP are not clear. Dietary fructose has been shown to enhance salt absorption in the intestine and kidney (Soleimani 2011). In the kidney, fructose acutely stimulates sodium hydrogen exchange 3 (NHE3) activity in isolated proximal tubules (Cabral et al. 2014). Fructose is theorized to potentially stimulate an increase in sympathetic activity and possibly increase sodium retention by inducing renin secretion (Abdulla et al. 2011). Yet, little to no research has been conducted to specifically investigate whether fructose stimulates sodium retention in the whole body during normal or high salt intake. Beierwaltes et al. (1982) performed cumulative sodium balance studies in young spontaneously hypertensive rats (SHR), and showed that SHR retained more sodium than age‐matched controls during the time phase when blood pressure increased. We found that fructose, but not glucose, significantly decreased urinary sodium excretion. By measuring Na intake and excretion, we found that during a normal salt diet there were no significant differences in sodium balance in rats on either control, 20% fructose, or 20% glucose diet. However, during the second week, when high salt diet was provided, sodium retention, as measured by cumulative sodium balance, increased to some extent in all three groups on high salt diet (F + HS, HS, G + HS) Further, rats on 20% fructose plus high salt retained twice the sodium than rats receiving high salt or 20% glucose plus high salt. These data suggest that the decreased sodium excretion in fructose‐fed rats is likely due to increased sodium retention during high salt intake. Presumably, if we had extended our study to 2–3 weeks, we would see all three high salt groups return into sodium balance, but the fructose plus high salt group maintaining a higher equilibrium pressure. To confirm that the renal sodium reabsorption mediated sodium retention, we also measured fecal sodium excretion in a subset of each experimental group. Total fecal sodium excretion was remarkably equal across groups on a normal salt diet, representing only 3–6% of total excreted sodium. When high salt was introduced, fecal sodium excretion did not increase, suggesting the intestine efficiently absorbed all of the additional sodium. Our data indicate that fructose or glucose did not decrease intestinal sodium reabsorption and the kidneys are responsible for regulating the excreted sodium. Our present study is novel in that we intentionally pair‐fed all groups of rats the same amount of food to maintain sodium intake constant between groups, and then measured baseline urinary sodium excretion prior to the introduction of 20% fructose or 20% glucose. This protocol allowed us to determine that fructose (but not glucose) decreased urinary sodium excretion in rats fed high salt diet.

Evidence substantiates that nitric oxide (NO) deficiency and oxidative stress contribute toward hypertension (Araujo and Wilcox 2014; Kone and Baylis 1997). Renal nitric oxide is an important regulator of urinary sodium excretion and has a natriuretic effect (Garvin et al. 2011; Herrera and Garvin 2005; Ortiz and Garvin 2002). Rats placed on 40% fructose for 2 weeks have significantly decreased expression of endothelial NO Synthase (Nishimoto et al. 2002). To estimate if fructose decreased renal NO production, we measured urinary NO_2_/NO_3_ excretion in control and fructose‐fed rats. Rats placed on a high salt diet have been shown to increase renal nitric oxide synthase expression (Mattson and Higgins 1996) and urinary NO_2_/NO_3_ excretion (Johnson et al. 2010). We also found that the high salt diet increased renal NO_2_/NO_3_ excretion in all high salt‐treated groups (F + HS, HS, G + HS). However, renal NO_2_/NO_3_ excretion in the 20% fructose + high salt group was significantly blunted compared to its high salt alone. These data do not provide pure cause and effect, but suggest that decreased renal nitric oxide availability may contribute to decreased sodium excretion and increased sodium retention in fructose fed rats.

Renin is the enzyme which is the rate‐limiting step in the formation of the hormone angiotensin II. Potentially, fructose may increase blood pressure by preventing the appropriate decrease in renin secretion typically caused by a high salt diet (Beierwaltes 2010). In protocol 2, in which rats had access to unlimited chow, a high salt diet significantly decreased PRA. However, the high salt‐induced suppression of PRA was blunted by addition of 20% fructose. These data suggest that fructose may blunt or prevent the expected suppression of PRA that should occur with high salt intake. This inappropriate normalization of PRA may contribute to increased blood pressure. Previously, studies blocking the renin‐angiotensin system have reported blunting high fructose‐induced hypertension (Chou et al. 2011, 2013).

Excessive fructose consumption (40–60%) can lead to insulin resistance, increased plasma insulin and triglyceride levels after 4 weeks (Reaven et al. 1990). However, we observed salt‐sensitive hypertension within 1 week of starting high salt. To validate that short‐term (2 weeks) 20% fructose‐induced salt‐sensitive hypertension occurred prior to the development of metabolic alterations, we measured fasting blood glucose, glucose tolerance, and triglycerides. Our 20% fructose diet minimally (but significantly) elevated blood glucose, which remained in a normo‐glycemic range, though the increase was modest. We performed glucose tolerance tests, and found that in fructose‐fed rats with or without high salt, glucose tolerance profiles were similar to control rats after 2 weeks on the diets. Total cholesterol and triglycerides were unchanged in our experimental groups except that triglycerides increased more than twofold (protocol 2) only in the F + HS group. These data suggest that just a single week of fructose plus high salt initiates some metabolic changes, which do not occur with the fructose alone or high salt alone. Thus, the rapid (days) onset of salt‐sensitive hypertension in our model precedes the development of major metabolic alterations.

In protocol 1, we found that the body weights were lower in non‐sugar supplemented rats, and this was a function of strict pair‐feeding to control salt intake. Our intent with pair‐feeding was to ensure consistent food intake to measure Na balance. Rats receiving 20% fructose and glucose supplemented water had access to more calories than control rats. Most studies involving fructose do not directly report the percentage of caloric intake consumed from fructose since varying concentrations in the drinking water result in differences in volume intake, and changes in salt content in the diet results in different food intake. We measured this, and found that when high salt diet was introduced, food consumption decreased in both fructose and glucose‐fed rats. Rats on glucose supplemented water received similar calories as rats on fructose, while hypertension was only present in the F + HS rats and not the G + HS rats. Overall, these data suggest that increased blood pressure was independent of body weight, and caloric intake, and caused by fructose but not glucose.

In summary, we report that, (1) Combining 20% fructose with a high salt diet caused blood pressure to rapidly increase; (2) The increase in blood pressure coincided with increased sodium retention and diminished sodium excretion during high salt diet. (3) Fructose impaired renal nitric oxide excretion, and (4) a fructose‐enriched diet blunted high salt‐induced inhibition of plasma renin activity. These factors may all contribute to the rapid onset of hypertension. Collectively, these results suggest fructose, when combined with high salt diet, can induce salt‐sensitive hypertension prior to onset of metabolic alterations, most likely by affecting renal Na handling. This study provides discrete physiological pathways, supporting the epidemiologic concerns about the excessive consumption of both fructose and sodium within the American diet.

## Conflict of Interest

The authors report no conflicts of interest in this work.
